# Pure Milk

**Published:** 1856-12

**Authors:** 


### PURE MILK.

A GLASS of it, how delicious *in New York!* But such a thing is not believed to be possible by multitudes of country people. Reader, we can furnish you with a glass of it any day in the year. We have been supplied with it ever since "*we went to housekeeping*" in Gotham. It is furnished us by Friend Gurney, of 203 West 19th street, no doubt a lineal kinsman of Joseph John, and Mrs. Fry. He gets it from "*Friends,*" too. "But how do you know it?" says one. We know it by various signs and symptoms, which are quite satisfactory to ourselves; as, for example, a gallon of it yields no sediment of dirt, sand, or anything else; second, it is of the right consistency, as measured with a scientific instrument, which "*gives*" the density of any fluid; the density of Mr. Gurney's dairy being compared with the yield of a hundred dollar cow, belonging to a neighbor of ours, when we lived at beautiful *Yonkers*, opposite the Palisades of the Hudson; thirdly, a large yield of thick rich cream is found on the surface of our daily supply, every morning; and all know that nothing but pure milk, from a healthy and well-fed cow, can spread over its surface, after a few hours rest, a layer of rich cream.

Now, these reasons are perfectly satisfactory to ourselves. A more conclusive test we offer to our inquisitors. Just look in the face of our little two-year-old "Alice Hall," who feeds on this milk twice a day, and has done so *for years!* She weighs now more than her elder brother "Bob," who was raised on Jersey milk, happening to be over there in the sand flats, when as yet he hadn't happened himself.

Our friend, Mr. P., went a hundred miles up the river, for the express purpose of getting pure milk for his child, and in a short time its entire scalp was covered with a disgusting eruption. On inquiry, it was found that it was not manufactured milk; it actually was yielded by the cow, but she was fed entirely on grain which had gone through the process of distillation. On making a change, the child speedily got well. *Let families look well into the quality of the milk which is supplied to them.* ☞ The brightest and most tidy-looking milk carts are often as suggestive as the "whitened sepulchres" of Scripture story.

